# Comprehensive Chemical Composition Evaluation of *Ziziphus jujuba* var. *spinosa* Germplasm Resources and Selection of Elite Cultivars for Seed, Pulp, and Leaf Utilization

**DOI:** 10.3390/molecules30224470

**Published:** 2025-11-19

**Authors:** Xiaochen Song, Yongqing Zhang, Longfei Zhang

**Affiliations:** College of Pharmacy, Shandong University of Traditional Chinese Medicine, Jinan 250355, China

**Keywords:** *Ziziphus jujuba* var. *spinosa*, UPLC-Q-Exactive Orbitrap MS/MS, HPLC-ELSD, entropy weighted-TOPSIS, active ingredient, germplasm quality

## Abstract

**Background**: *Ziziphus jujuba Mill.* var. *spinosa* (Bunge) Hu ex H. F. Chow. (ZS) is a valuable plant with diverse economic applications, as all its organs contain bioactive secondary metabolites. The seeds, known as Suanzaoren in traditional Chinese medicine, are utilized as both a medicinal and edible resource, while the fruit pulp and leaves serve as significant raw materials in the food industry. Increasing market demand for Suanzaoren has led to expanded cultivation, though current production practices emphasize seed utilization, resulting in the underutilization of pulp and leaf tissues. In agricultural systems, developing elite varieties is an effective strategy for enhancing crop yield and quality. Breeding initiatives should establish specific objectives aligned with particular end uses, such as seed, pulp, or leaf production. Germplasm serves as the foundational material for breeding programs, so its selection must correspond to intended applications. Evaluating existing germplasm resources based on chemical composition profiles will provide a basis for developing improved ZS varieties. **Objective**: This study aimed to systematically compare the characteristic chemical composition in the seeds, pulp, and leaves of ZS. By quantifying key chemical components—such as flavonoid glycosides and saponins in seeds, organic acids and phenolic compounds in pulp, and flavonol glycosides and phenolic acids in leaves—we evaluated the quality of ZS germplasm resources. The resulting compositional profiles provide a concrete basis for selecting and breeding elite cultivars tailored to specific end uses, including seed, pulp, or leaf production. **Methods**: Chemical characterization was performed using ultra-high-performance liquid chromatography coupled with hybrid quadrupole-orbitrap mass spectrometry (UPLC-Q-Exactive Orbitrap MS/MS). Quantitative analysis of chemical composition was conducted using high-performance liquid chromatography with evaporative light scattering detection (HPLC-ELSD). Multivariate statistical analyses—including principal component analysis (PCA), orthogonal partial least squares discriminant analysis (OPLS-DA), and entropy-weighted technique for order preference by similarity to an ideal solution (entropy-weighted TOPSIS)(EWT)—were employed for comprehensive data evaluation. **Results**: A comprehensive phytochemical analysis of *Ziziphi spinosae* (ZS) was conducted, identifying 144 distinct compounds across the seeds, pulp, and leaves. Of these, 114 were found in the seeds, 84 in the leaves, and 79 in the pulp. The seeds were particularly rich in flavonoid glycosides, such as spinosin and 6‴-feruloylspinosin, as well as saponins like jujuboside A and B. The pulp was dominated by organic acids, including citric acid, and phenolic compounds, while the leaves were abundant in flavonol glycosides, including rutin, and phenolic acids such as isochlorogenic acid B. Based on the chemical composition profiles, the ZS germplasms were evaluated for specific applications. ZS24, ZS22, and ZS3 were identified as the most suitable for seed production, ZS3, ZS6, and ZS9 for pulp utilization, and ZS20, ZS3, and ZS18 for leaf-based applications. With respect to the integrated utilization of multiple plant parts (roots, stems, and leaves), ZS6, ZS3, and ZS24 demonstrated the highest potential. **Conclusions**: The identification of superior germplasm resources provides strategic direction for the breeding of elite ZS cultivars. These findings will enable the comprehensive utilization of ZS plant resources and support the high-quality development of related industries.

## 1. Introduction

*Ziziphus jujuba Mill.* var. *spinosa* (Bunge) Hu ex H. F. Chow., belonging to the Rhamnaceae family, holds significant economic and medicinal value and is found across Asia, Europe, and the Americas [1]. The entire plant possesses medicinal and edible utility: its seed, known as Suanzaoren in traditional Chinese medicine, is a medicinal food homolog. It is sweet in taste and neutral in nature, and is used to nourish the liver, calm the heart, arrest sweating, and promote fluid production, primarily for treating conditions such as insomnia, palpitations, night sweats, and thirst due to fluid deficiency. It also shows potential in alleviating anxiety, depression, and Alzheimer’s disease [2,3,4]. The pulp, rich in vitamins, trace elements, and various bioactive compounds, can tonify Qi and strengthen the spleen, improve complexion, and is consumed fresh or used as a food additive [5,6,7]. The leaves, valued for their nutritional and bioactive content, are used to make tea and are renowned as the “Oriental Sleep Leaf” for their preventive effects against coronary heart disease [8,9].

Modern research has systematically elucidated its chemical constituents and pharmacological activities. Chemically, the seeds are characterized by flavonoid-C-glycosides (e.g., spinosin), dammarane-type triterpenoid saponins (e.g., jujubosides A and B), and cyclic peptide alkaloids [10,11,12]. The pulp is marked by organic acids, polyphenols, and cyclic nucleotides, while the leaves are a rich source of flavonol glycosides (e.g., rutin) and phenolic acids [13,14,15]. Pharmacologically, the sedative-hypnotic effects of the seeds are largely attributed to their saponins and flavonoids, while the cyclic peptide alkaloids are associated with anti-anxiety activity [16,17]. In quality control, alongside the pharmacopoeia-stipulated HPLC methods, UPLC-Q-TOF-MS/MS-based metabolomics has emerged as a vital tool for precise compound identification, germplasm differentiation, and comprehensive quality assessment [2,18,19].

The recent increase in the market value of ZS seeds has driven a focus on seed production, leading to the disposal of significant amounts of pulp and leaf biomass as waste, raising concerns about resource inefficiency and environmental impact [20,21]. This underutilization reveals a key deficiency in the ZS value chain, underscoring the need for cultivars optimized for the comprehensive use of all plant components. This study integrated UPLC-Q-Exactive Orbitrap MS/MS and HPLC-ELSD technologies to systematically profile metabolites, conduct comparative analysis, and evaluate the comprehensive chemical quality of three main parts (seeds, leaves, and pulp) from 26 different *Ziziphus jujuba* var. *spinosa* germplasms for the first time. Principal component analysis (PCA), orthogonal partial least squares-discriminant analysis (OPLS-DA), and the entropy-weighted TOPSIS model were employed for multi-indicator decision-making. The research aims to provide a more comprehensive and reliable chemical basis for selecting superior germplasms tailored to specific application targets, thereby holding significant practical implications for promoting high-quality development in the *Ziziphus jujuba* var. *spinosa* industry.

## 2. Results

### 2.1. Identification and Characterization of Chemical Composition in ZS Tissues

A comprehensive phytochemical analysis of ZS seeds, pulp, and leaves was performed using UPLC-Q-Exactive Orbitrap MS/MS, focusing on retention time, exact mass, and MS/MS fragmentation patterns. A total of 144 compounds were identified, revealing distinct metabolic profiles across tissues: seeds showed the greatest compositional diversity with 114 compounds, compared to 84 in leaves and 79 in pulp (Table 1). All metabolite identifications were assigned a Level 2 confidence rating based on the Metabolomics Standards Initiative (MSI) framework. The chemical composition were categorized into five primary classes: flavonoids (40), terpenoids (21), organic acids (34), alkaloids (15), and amino acids (12), along with trace compounds such as lignans, coumarins, nucleosides, and amides. This detailed compositional profile provides a crucial basis for further quantitative analysis of key chemical composition.

### 2.2. Quantification of Major Chemical Composition Across ZS Germplasms

#### 2.2.1. Validation of the Quantitative Analytical Method

The results of the methodological validation for quantitative analysis of major chemical composition in ZS seeds, pulp, and leaves are presented in Table A1, Appendix A. Excellent linearity (R^2^ > 0.999) was observed for all calibration curves across the tested concentration ranges. The relative standard deviation (RSD) values for precision, repeatability, and stability were all below 3%. Average recoveries ranged from 97.50% to 102.98%, with RSD values below 3.00%, confirming the validity and reliability of the established method.

#### 2.2.2. Content of Major Chemical Composition in Seeds

The contents of six major chemical composition—cryptochlorogenic acid, caffeic acid, spinosin, 6‴-feruloylspinosin, jujuboside A, and jujuboside B—were quantified in seeds across 26 ZS germplasms (Table A2). Considerable variation was observed among germplasms, with concentration ranges and coefficients of variation (CV) as follows: cryptochlorogenic acid (0.0394–0.1818 mg/g; CV = 43.38%), caffeic acid (0.1842–2.1300 mg/g; CV = 46.62%), spinosin (0.3413–1.8418 mg/g; CV = 35.32%), 6‴-feruloylspinosin (1.4580–5.5312 mg/g; CV = 36.00%), jujuboside A (0.2396–0.9615 mg/g; CV = 36.45%), and jujuboside B (0.1250–0.1942 mg/g; CV = 11.98%). The highest content of each compound was detected in ZS15 (cryptochlorogenic acid), ZS24 (caffeic acid), ZS4 (spinosin), ZS22 (6‴-feruloylspinosin), ZS24 (jujuboside A), and ZS4 (jujuboside B), while the lowest values were observed in ZS20, ZS26, ZS21, ZS20, ZS21, and ZS8, respectively. Germplasm ZS24 exhibited the highest total content of the six chemical composition, whereas ZS21 showed the lowest. Notably, ZS24 also contained the highest levels of spinosin and jujuboside A, two compounds of significant pharmacological relevance, highlighting its potential as a superior germplasm for seed applications.

#### 2.2.3. Content of Major Chemical Composition in Pulp

Quantitative analysis of seven major chemical composition in the pulp—citric acid, gallic acid, catechin, caffeic acid, ferulic acid, rutin, and quercetin-3-*O*-glucoside—was performed across the 26 germplasms (Table A3). The content ranges and corresponding coefficients of variation were as follows: citric acid (1.4692–20.4395 mg/g; CV = 53.70%), gallic acid (0.0326–0.2219 mg/g; CV = 41.11%), catechin (0.0406–0.1485 mg/g; CV = 30.44%), caffeic acid (0.0017–0.0181 mg/g; CV = 52.41%), ferulic acid (0.0006–0.0081 mg/g; CV = 74.87%), rutin (0.0280–0.3270 mg/g; CV = 58.92%), and quercetin-3-*O*-glucoside (0.0025–0.0148 mg/g; CV = 28.00%). The germplasms with the highest individual compound content were ZS1 (citric acid), ZS26 (gallic acid), ZS3 (catechin), ZS3 (caffeic acid), ZS11 (ferulic acid), ZS6 (rutin), and ZS3 (quercetin-3-*O*-glucoside). The lowest values were identified in ZS23, ZS16, ZS7, ZS13, ZS3, ZS26, and ZS16, respectively. ZS1 displayed the highest total content of the seven compounds, while ZS23 exhibited the lowest.

#### 2.2.4. Content of Major Chemical Composition in Leaves

Thirteen major chemical compositions were quantified in the leaves: citric acid, neochlorogenic acid, catechin, caffeic acid, rutin, quercetin-3-*O*-glucoside, kaempferol-3-*O*-rutinoside, isochlorogenic acid B, astragalin, quercetin, jujuboside A, jujuboside B, and kaempferol (Table A4). The concentrations and variability among germplasms were as follows: citric acid (0.3820–1.0655 mg/g; CV = 30.81%), neochlorogenic acid (0.0046–0.0478 mg/g; CV = 55.73%), catechin (0.0088–0.1344 mg/g; CV = 93.65%), caffeic acid (0.0126–0.1302 mg/g; CV = 51.23%), rutin (2.8404–18.8823 mg/g; CV = 46.99%), quercetin-3-*O*-glucoside (0.2564–0.8187 mg/g; CV = 30.71%), kaempferol-3-*O*-rutinoside (0.0598–0.7043 mg/g; CV = 57.13%), isochlorogenic acid B (0.0024–3.1403 mg/g; CV = 92.25%), astragalin (0.0104–0.1644 mg/g; CV = 62.53%), quercetin (0.0910–0.3637 mg/g; CV = 39.21%), jujuboside A (0.1028–0.8472 mg/g; CV = 52.26%), jujuboside B (0.1099–0.4504 mg/g; CV = 35.05%), and kaempferol (0.2105–1.5576 mg/g; CV = 61.45%). The highest contents were found in ZS20 (citric acid), ZS4 (neochlorogenic acid), ZS3 (catechin), ZS6 (caffeic acid), ZS6 (rutin), ZS20 (quercetin-3-*O*-glucoside), ZS6 (kaempferol-3-*O*-rutinoside), ZS18 (isochlorogenic acid B), ZS20 (astragalin), ZS20 (quercetin), ZS11 (jujuboside A), ZS13 (jujuboside B), and ZS20 (kaempferol). The lowest values corresponded to ZS22, ZS3, ZS12, ZS11, ZS7, ZS6, ZS7, ZS12, ZS22, ZS7, ZS15, ZS5, and ZS7, respectively. Germplasm ZS6 exhibited the highest total content of the thirteen compounds, while ZS7 showed the lowest.

### 2.3. Quality Evaluation of ZS Germplasm

ZS possesses significant economic value due to its well-documented medicinal applications across multiple plant organs. The seeds, commercially known as Suanzaoren, are established therapeutic agents and nutritional supplements. Similarly, the pulp is directly consumable or serves as a key raw material in food processing, while the leaves are traditionally used in herbal tea production. Consequently, quality evaluation of ZS germplasm must be rigorously aligned with these specific end-use applications to ensure functional efficacy and economic viability.

#### 2.3.1. Quality Evaluation of Seed-Use ZS Germplasm Based on Major Seed Chemical Composition

The clinical effectiveness of Suanzaoren is fundamentally determined by the composition and concentration of its chemical composition. Therefore, quantifying these constituents is essential for robust quality assessment of seed-oriented ZS germplasm.

##### Hierarchical Cluster Analysis (HCA)

**Figure A1:**
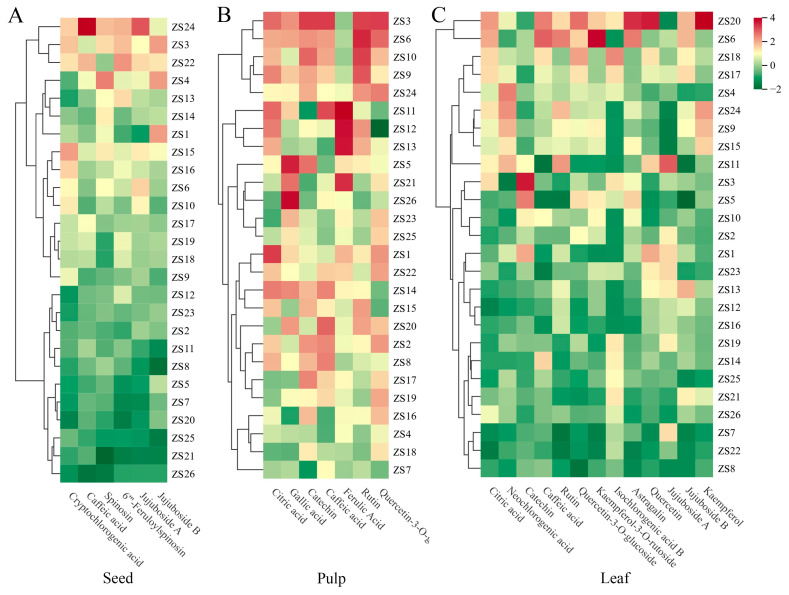
Hierarchical cluster analysis (HCA) heatmap of different ZS germplasms based on major chemical composition in seeds (**A**), pulp (**B**), and leaves (**C**).

HCA was conducted using the concentrations of six major chemical composition in seeds as classification variables, resulting in a cluster heatmap that delineates the 26 ZS germplasms (Figure A1A). In this heatmap, color intensity directly corresponds to relative abundance, with red and green indicating high and low levels, respectively. Based on distinct phytochemical profiles, the germplasms were decisively segregated into three groups: A, B, and C. Group A, containing the highest chemical composition, included ZS24, ZS3, and ZS22. Group B, with intermediate levels, comprised ZS4, ZS13, ZS14, ZS1, ZS15, ZS16, ZS6, ZS10, ZS17, ZS19, ZS18, and ZS9. Group C, showing the lowest accumulation, contained the remaining accessions. Each cluster demonstrated strong internal consistency, validating the classification reliability.

##### Principal Component Analysis (PCA)

**Figure 1:**
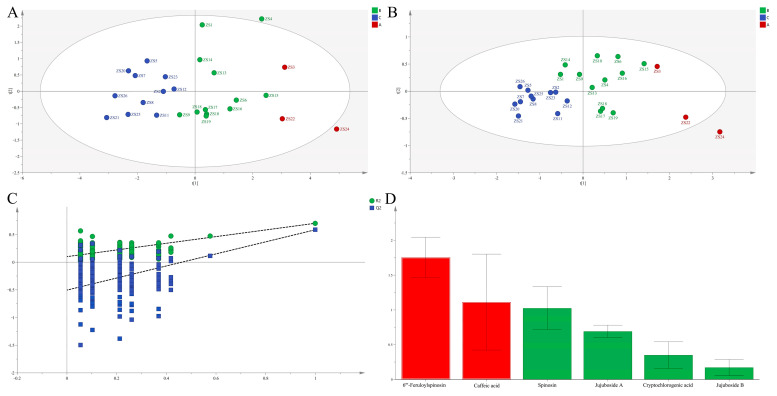
PCA score plots (**A**) and OPLS-DA score plots (**B**), permutation validation (**C**), and variable importance in projection (VIP) (**D**) analysis of different ZS germplasms based on chemical composition in seeds.

To further verify the cluster structure, PCA was performed using the same six chemical composition. Data processed through SIMCA 14.1 produced two principal components explaining R^2^X [1] = 0.680 and R^2^X [2] = 0.128 of total variance. The resulting score plot (Figure 1A) conclusively affirmed the HCA-derived classification, similarly separating germplasms into three discrete groups, thereby reinforcing the robustness of the identified clusters.

##### Orthogonal Partial Least Squares Discriminant Analysis (OPLS-DA)

To minimize within-group variation and identify discriminatory chemical composition, OPLS-DA was applied following PCA. The model exhibited strong explanatory power, with R^2^X = 0.985, R^2^Y = 0.540, and Q^2^ = 0.324 (Figure 1B). Clear group separation in the score plot confirmed significant inter-germplasm heterogeneity in chemical composition. Model validity was unequivocally established through a rigorous 200-iteration permutation test, which effectively ruled out overfitting. VIP analysis definitively identified two key discriminatory compounds—6‴-feruloylspinosin and caffeic acid—both with VIP values exceeding 1.

##### Entropy-Weighted TOPSIS Analysis (EWT)

**Figure 2:**
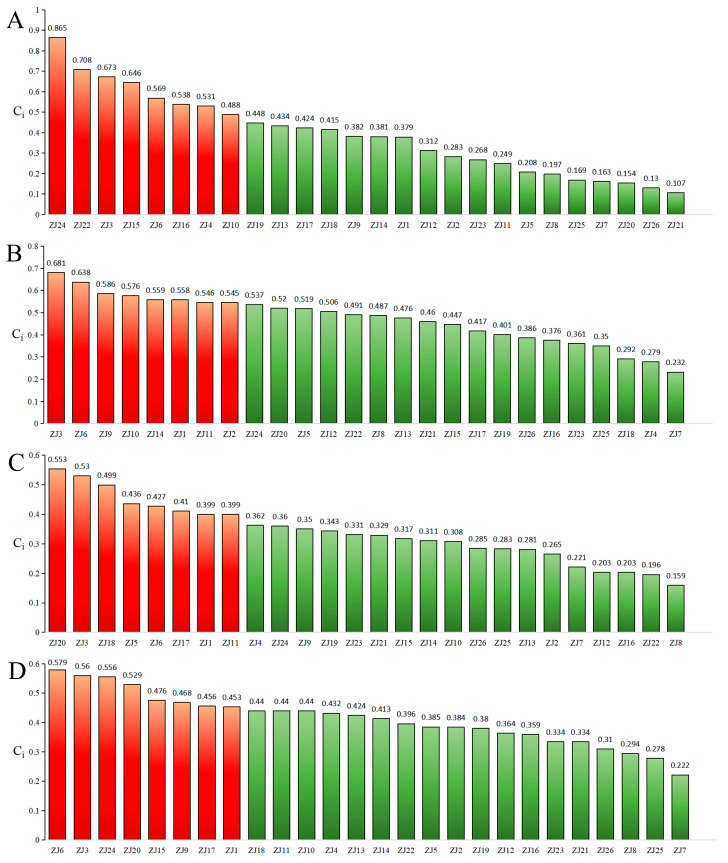
Comprehensive quality ranking of ZS germplasms based on chemical composition ((**A**): seed-use; (**B**): pulp-use; (**C**): leaf-use; (**D**): comprehensive utilization).

Significant chemotypic diversity was observed among the 26 germplasms based on the six major chemical composition. To comprehensively evaluate overall quality, an EWT approach was employed. Weighting coefficients were systematically derived from compound abundance data using the entropy method. Subsequent normalization of the raw data matrix was performed according to Equation (1), as all indicators were beneficial. Indicator proportions and respective weights were calculated using Equations (2)–(5), followed by the construction of a weighted normalization matrix. Positive and negative ideal solutions were definitively established using Equations (6) and (7), respectively. Euclidean distances to these ideals and relative closeness coefficients (Cᵢ) were calculated using Equations (8)–(10), with higher Cᵢ values unequivocally indicating superior germplasm quality. The eight elite accessions—ZS24, ZS22, ZS3, ZS15, ZS6, ZS16, ZS4, and ZS10—achieved a mean Cᵢ of 0.627, confirming their superior phytochemical quality. These results demonstrate strong concordance with the HCA-based classification (Figure 2A).

(1) $$Y_{ij}=\frac{X_{ij}-\min\left(x_{j}\right)}{\max\left(x_{j}\right)-\min\left(x_{j}\right)}$$

(2) $$p_{ij}=\frac{y_{ij}}{\sum_{i=1}^{n}y_{ij}}\;\;$$

(3) $$e_{j}=-\frac{1}{\ln\left(n\right)}\sum_{i=1}^{n}p_{ij}lnp_{ij}\;\;\;\;\;\;\;\;$$

(4) $$d_{j}=1-e_{j}$$

(5) $$w_{j}=\frac{d_{j}}{\sum_{j=1}^{n}d_{j}}\;\;\;$$

(6) $$A^{+}=\left\{\max\left(A_{ij}\right)|j=1,2,\ldots ,m\right\}\;$$

(7) $$A^{-}=\left\{\min\left(A_{ij}\right)|j=1,2,\ldots ,m\right\}\;\;$$

(8) $$D_{i}^{+}=\sqrt{\sum_{j=1}^{m}{\left(A_{ij}-A^{+}\right)}^{2}}\;\;\;\;$$

(9) $$D_{i}^{-}=\sqrt{\sum_{j=1}^{m}{\left(A_{ij}-A^{-}\right)}^{2}}\;$$

(10) $$C_{i}=\frac{D_{i}^{+}}{D_{i}^{+}+D_{i}^{-}}\;$$

*Y*_*i*j_: Data normalization; $X_{ij}$: the original data; $p_{ij}$: the characteristic weight; $e_{j}$: the entropy value; $d_{j}$: the utility value; *w*_*j*_: the weight; $A^{+}$: the positive ideal solution; $A^{-}$: the negative ideal solution; $D_{i}^{+}$: the positive ideal distance; $D_{i}^{-}$: the negative ideal distance; $C_{i}$: the closeness coefficient.

#### 2.3.2. Quality Evaluation of Pulp-Use ZS Germplasm Based on Major Chemical Composition in Pulp

The health benefits of ZS pulp consumption are directly attributable to its chemical composition. Thus, precise quantification of these compounds is crucial for evaluating pulp-use germplasm quality.

##### HCA

HCA conducted using seven major chemical compositions in pulp generated a cluster heatmap of germplasms (Figure A1B), revealing substantial inter-germplasm variation. Germplasms were classified into four groups (D–G). Group D, with the highest content, included ZS3, ZS6, ZS10, ZS9, and ZS24; Group E, showing moderately high levels, contained ZS11, ZS12, and ZS13; Group F demonstrated intermediate accumulation (ZS5, ZS21, ZS26, ZS23, ZS25); Group G, with minimal content, comprised the remaining accessions.

##### PCA

**Figure 3:**
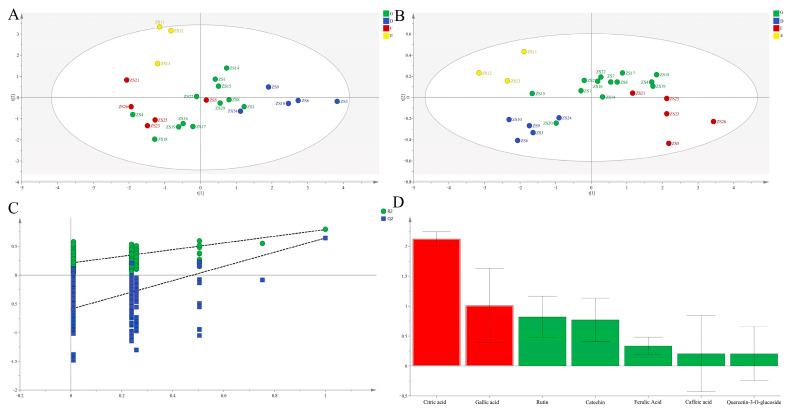
PCA score plots (**A**) and OPLS-DA score plots (**B**), permutation validation (**C**), and variable importance in projection (VIP) (**D**) analysis of different ZS germplasms based on chemical composition in pulp.

PCA based on the seven compounds yielded R^2^X [1] = 0.350 and R^2^X [2] = 0.237 (Figure 3A), robustly corroborating the four-group structure identified by HCA.

##### OPLS-DA

Subsequent OPLS-DA modeling demonstrated excellent goodness-of-fit (R^2^X = 1.000, R^2^Y = 0.735, Q^2^ = 0.459; Figure 3B), revealing significant inter-group phytochemical differences. Model robustness was confirmed through permutation testing. Citric acid and gallic acid (VIP >1) were definitively identified as key discriminators.

##### EWT

EWT analysis clearly identified ZS3, ZS6, ZS9, ZS10, ZS14, ZS1, ZS11, and ZS2 as superior germplasms (mean Cᵢ = 0.586), consistent with HCA classification (Figure 2B).

#### 2.3.3. Quality Evaluation of Leaf-Use ZS Germplasm Based on Major Chemical Composition in Leaves

ZS leaves are utilized in functional tea production, where chemical composition serve as primary quality determinants. Therefore, their accumulation levels are a critical criterion for evaluating leaf-use germplasm.

##### HCA

HCA using 13 leaf chemical composition effectively classified germplasms into three distinct groups (H–J) (Figure A1C). Group H (highest content) included ZS20 and ZS6; Group I (intermediate) comprised eight accessions, included ZS18, ZS17, ZS4, ZS24, ZS9, ZS15, ZS11, and ZS1.; Group J (lowest) contained the remainder.

##### PCA

**Figure 4:**
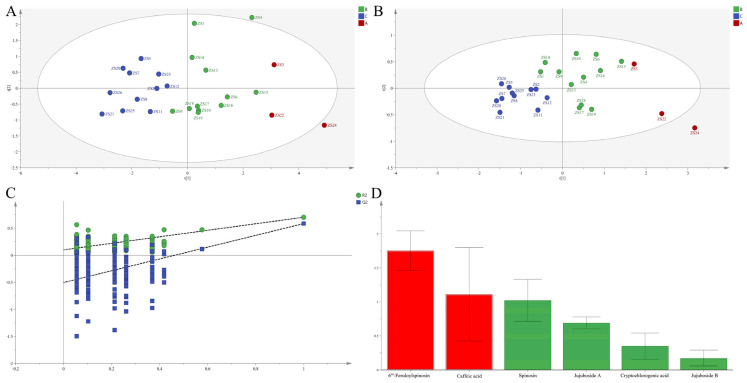
PCA score plots (**A**) and OPLS-DA score plots (**B**), permutation validation (**C**), and variable importance in projection (VIP) (**D**) analysis of different ZS germplasms based on chemical composition in leaves.

PCA (R^2^X [1] = 0.406, R^2^X [2] = 0.138; Figure 4A) conclusively affirmed the three-group classification.

##### OPLS-DA

OPLS-DA (R^2^X = 1.000, R^2^Y = 0.870, Q^2^ = 0.226; Figure 4B) confirmed significant inter-group differentiation. Rutin, isochlorogenic acid B, and kaempferol-3-*O*-rutinoside (VIP > 1) were identified as discriminatory markers.

##### EWT

TOPSIS analysis definitively identified ZS20, ZS3, ZS18, ZS5, ZS6, ZS17, ZS1, and ZS11 as elite germplasms (mean Cᵢ = 0.457), aligning with HCA groupings (Figure 2C).

#### 2.3.4. Comprehensive Quality Evaluation of ZS Germplasm Integrating Major Chemical Composition from Seeds, Pulp, and Leaves

Conventional germplasm evaluation typically focuses on single-organ utilization, primarily seeds. However, exclusive reliance on seeds may lead to diminished economic returns with expanded cultivation, highlighting the necessity of whole-plant valorization. A comprehensive quality assessment using EWT incorporated chemical composition from all three organs as positive indicators (Figure 2D). The top-eight germplasms—ZS6, ZS3, ZS24, ZS20, ZS15, ZS9, ZS17, and ZS1 (mean Cᵢ = 0.510)—were conclusively identified as possessing superior holistic quality, underscoring their significant potential in breeding programs aimed at multi-purpose crop development.

## 3. Discussion

*Ziziphus jujuba* var. *spinosa* is an economically significant crop with well-established medicinal value. While its seeds (Suanzaoren) are a cornerstone of traditional Chinese medicine, the pulp and leaves also contain potentially bioactive metabolites, offering substantial potential for edible and medicinal applications. This study employed an integrated approach using UPLC-Q-Exactive Orbitrap MS/MS and HPLC-ELSD to systematically characterize the chemical constituents in these three plant parts. A total of 144 compounds were identified, revealing the highest phytochemical diversity in the seeds (114 compounds), followed by the leaves (84 compounds) and pulp (79 compounds). These findings provide a chemical basis confirming the seeds as the primary medicinal component, while also highlighting the substantial potential for broader utilization of the pulp and leaves.

The three plant parts exhibited distinct chemical profiles supporting their differential applications: Seeds were rich in flavonoid glycosides and saponins, with 6‴-feruloylspinosin being the most abundant (1.46–5.53 mg/g). The feruloyl moiety in this compound enhances blood–brain barrier permeability [1,53] and, together with structurally related compounds like spinosin, contributes to the sedative, hypnotic, anxiolytic, and cognitive-enhancing effects of Suanzaoren, including documented potential in Alzheimer’s disease management [54,55]. Pulp was characterized by high levels of organic acids and polyphenols. Its elevated citric acid content imparts a characteristic sourness while providing preservative, antioxidant, and flavor-enhancing properties [56]. Phenolic compounds further strengthen its antioxidant capacity, supporting its traditional use as a food additive, acidulant, and nutritional supplement rich in polysaccharides, amino acids, and vitamins [57]. Leaves contained substantial amounts of flavonol glycosides, known to confer anti-inflammatory, anti-allergic, and antioxidant effects, in addition to enhancing capillary resilience [58,59,60].

## 4. Materials and Methods

### 4.1. Materials

#### 4.1.1. Plant Material

Samples of ZS were collected from a standardized cultivation base operated by Shandong Zhongchang Yuan Investment Group Co., Ltd., located in the Yellow River Delta Agricultural High-tech Industrial Demonstration Zone, Dongying City, Shandong Province, China. This site was selected to ensure environmental consistency and genetic representativeness of the samples.

A total of twenty-six germplasms (labeled ZS1 to ZS26) were selected based on observable phenotypic variation among the plants. On 12 August 2024, fruits and leaves were randomly harvested from each germplasm to reduce sampling bias. Samples were rinsed with purified water and air-dried. The pulp was manually separated from the endocarp, and seeds were extracted by mechanically cracking the stones. Seeds, pulp, and leaves were dried to constant weight at 50 °C, ground into a fine powder with an electric grinder, sieved through a 60-mesh sieve, and stored in a desiccator for further analysis.

Taxonomic authentication was authoritatively performed by Professor Yongqing Zhang of Shandong University of Traditional Chinese Medicine, which confirmed all germplasms as *Ziziphus jujuba Mill.* var. *spinosa* (Bunge) Hu ex H. F. Chow. (family Rhamnaceae). These accessions represent distinct germplasms primarily distinguished by macroscopic morphological differences in plant and fruit characteristics.

#### 4.1.2. Chemicals and Reagents

All reference compounds—including cryptochlorogenic acid (1), caffeic acid (2), spinosin (3), 6‴-feruloylspinosin (4), jujuboside A (5), jujuboside B (6), citric acid (7), gallic acid (8), catechin (9), ferulic acid (10), rutin (11), quercetin-3-*O*-glucoside (12), neochlorogenic acid (13), kaempferol-3-*O*-rutoside (14), isochlorogenic acid B (15), astragalin (16), quercetin (17), and kaempferol (18)—were sourced from Shanghai Yuanye Biotechnology Co., Ltd. (Shanghai, China). Each compound was rigorously certified to possess a purity of ≥98% through HPLC analysis, thereby ensuring reliability and reproducibility of analytical results.

High-purity formic acid (guaranteed reagent grade) and HPLC-grade acetonitrile were procured from Thermo Fisher Scientific (Waltham, MA, USA), while purified water was supplied by Watsons Food & Beverage Co., Ltd. (Guangzhou, China).

### 4.2. Methods

#### 4.2.1. Preparation of Sample Solutions

Accurately weighed powdered samples (1.0 g) were transferred into sealed conical flasks and combined with 10 mL of methanol. The flasks were reweighed to account for any solvent loss, then subjected to optimized ultrasonication (KQ-500DE CNC Ultrasonic Cleaner, Jiangsu, China) at 30 °C for 60 min (500 W, 40 kHz) to maximize compound extraction. After cooling to room temperature, the total weight was recorded, and methanol was added to compensate for any solvent loss, ensuring volumetric accuracy. The mixtures were thoroughly vortexed to achieve homogeneity, centrifuged (SorvallST8R, Thermo Scientific, Waltham, MA, USA) at 12,000× *g* r/min for 15 min to remove particulates, and the supernatants were filtered through 0.22 μm membranes to eliminate residual impurities prior to chromatographic analysis.

#### 4.2.2. Preparation of Standard Solutions

Individual stock solutions were prepared by dissolving accurately weighed reference standards in methanol, followed by dilution to volume in calibrated volumetric flasks to ensure precise concentration levels. The concentrations of each stock solution were established as follows (in mg/mL): (1) 0.960, (2) 1.200, (3) 0.990, (4) 1.090, (5) 0.960, (6) 0.950, (7) 1.292, (8) 0.980, (9) 0.985, (10) 1.075, (11) 2.250, (12) 1.020, (13) 1.115, (14) 1.130, (15) 1.080, (16) 1.090, (17) 1.120, and (18) 1.030.

Working standard solutions were prepared by diluting aliquots of stock solutions with methanol to achieve concentrations within the linear range of detection. All solutions were stored at 4 °C and filtered through a 0.22 μm membrane before injection to prevent column contamination and ensure analytical consistency.

#### 4.2.3. UPLC-Q-Exactive-Orbitrap-MS/MS Conditions for Qualitative Analysis

Chromatographic separation was performed using a Thermo Vanquish-Orbitrap Exploris 120 system equipped with a HALO C18 column (2.1 mm × 100 mm, 2.7 μm; Agilent, Santa Clara, CA, USA). The mobile phase consisted of 0.1% formic acid in water (phase A) and 0.1% formic acid in acetonitrile (phase B), delivered at a constant flow rate of 0.3 mL/min with the column temperature maintained at 30 °C. The gradient elution profile was programmed as follows: 0–3 min, 5–11% B; 3–3.5 min, 11–14% B; 3.5–11.5 min, 14–33% B; 11.5–12 min, 33–36% B; 12–15 min, 36–44% B; 15–18 min, 44–74% B; 18–26 min, 74–95% B; 26–28 min, 95% B.

Ionization was performed using a heated electrospray ionization (HESI) source in both positive and negative ion modes. The parameters were optimized for sensitivity and spectral accuracy: capillary voltage at 350 V, capillary and vaporizer temperatures at 350 °C, mass range *m*/*z* 80–121, resolution at 70,000, S-Lens RF level at 55, sheath gas flow at 50 arb, and auxiliary gas flow at 10 arb.

**Figure 5:**
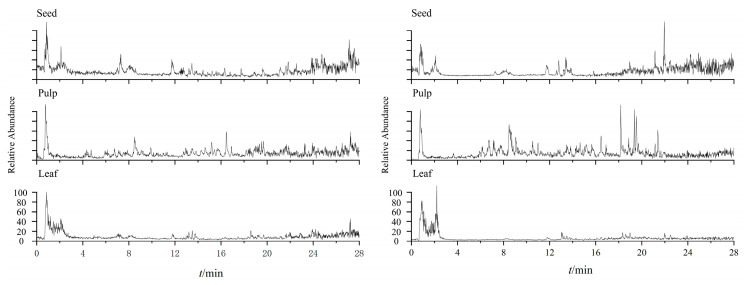
Base peak chromatograms (BPCs) of seed, pulp, and leaf extracts from S acquired in positive (**left**) and negative (**right**) ion modes.

Figure 5 comprehensively illustrates representative chromatograms of seed, pulp, and leaf extracts in both ionization modes, providing a clear basis for compound identification.

#### 4.2.4. HPLC-ELSD Conditions for Quantitative Analysis

Quantitative analysis was conducted using an Agilent 1260 Infinity II HPLC system coupled with an Agilent 1290 Infinity II Evaporative Light Scattering Detector (ELSD). Separation was achieved on a ZORBAX SB-C18 column (4.6 mm × 250 mm, 5 μm; Agilent, Santa Clara, CA, USA) maintained at 30 °C, with a mobile phase flow rate of 1.0 mL/min. The mobile phase comprised 0.1% formic acid in water (A) and 0.1% formic acid in acetonitrile (B). The gradient elution conditions were specifically optimized for each tissue type: Seeds: 0–5 min, 15–25% B; 5–8 min, 25–36% B; 8–9 min, 36–39% B; 9–17 min, 39–42% B; 17–18 min, 42–43% B. Pulp: 0–15 min, 5–13% B; 15–30 min, 13–15% B; 30–45 min, 15–20% B. Leaves: 0–11 min, 5–25% B; 11–12 min, 25–29% B; 12–20 min, 29–50% B; 20–24 min, 50–73% B.

**Figure 6:**
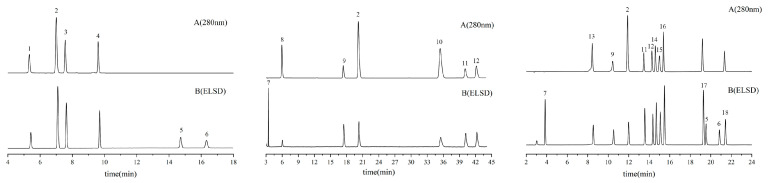
Representative HPLC-ELSD chromatogram of the mixed reference standard solution.

**Figure 7:**
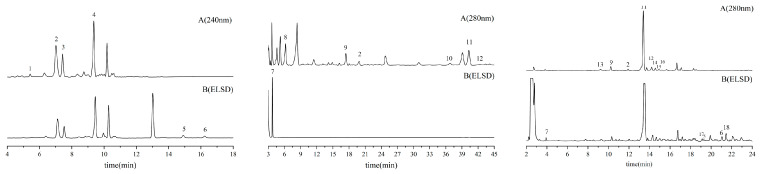
Representative HPLC-ELSD chromatograms of seed (**left**), pulp (**middle**), and leaf (**right**) extracts from ZS under detection conditions: A. 240 nm and B. ELSD.^1^ Cryptochlorogenic acid, ^2^ Caffeic acid, ^3^ Spinosin, ^4^ 6‴-Feruloylspinosin, ^5^ Jujuboside A, ^6^ Jujuboside B,^7^ Citric acid, ^8^Gallic acid, ^9^ Catechin, ^10^ Ferulic Acid, ^11^ Rutin, ^12^ Quercetin-3-*O*-glucoside, ^13^ Neochlorogenic acid, ^14^ Kaempferol-3-*O*-rutoside, ^15^ Isochlorogenic acid B, ^16^ Astragalin, ^17^ Quercetin, ^18^ Kaempferol.

Chromatograms for reference standards and sample extracts are explicitly provided in Figure 6 and Figure 7, respectively, facilitating direct comparative analysis.

#### 4.2.5. Validation of the Quantitative Method

The analytical method was comprehensively validated for linearity, precision, stability, repeatability, and recovery in accordance with ICH guidelines. A six-point calibration curve was constructed through serial dilution of a mixed standard solution, and linear regression analysis was performed by plotting peak area (Y) against concentration (X), consistently yielding correlation coefficients (R^2^) exceeding 0.999. Precision was unequivocally demonstrated through six consecutive injections of the same standard, resulting in RSD values below 2%. Stability was assessed by analyzing identical samples over 0, 2, 4, 8, 12, and 24 h, confirming analyte integrity under storage conditions. Repeatability was validated using six independently prepared sample replicates, with RSD values consistently under 3%. Recovery tests were performed using spiked samples in six replicates, yielding recovery rates between 97.5% and 102.5%, calculated as:

Recovery (%) = (measured amount in spiked sample − original amount)/amount added × 100%

#### 4.2.6. Statistical Analysis

Qualitative data were analyzed using Xcalibur™ 4.3 software (Thermo Fisher Scientific, Waltham, MA, USA) with a mass accuracy tolerance of ≤10 ppm. Multivariate analyses, including PCA and OPLS-DA, were performed in SIMCA 14.1 (Umetrics AB, Umea, Sweden). HCA and heatmap visualization were carried out using the Metware Cloud platform (https://cloud.metware.cn, accessed on 18 July 2025). EWT was executed in Microsoft Excel 2021.

## 5. Conclusions

Building on the distinct chemical profiles established in this study, elite germplasms were successfully identified for specific applications through multi-component quantification. The optimal germplasms for different utilization purposes were determined as follows: ZS24, ZS3, and ZS22 were optimal for seed production; ZS3, ZS6, and ZS9 for pulp utilization; and ZS20, ZS3, and ZS18 for leaf applications. For integrated use of all three organs, ZS6, ZS3, and ZS24 demonstrated superior performance.

The recent market-driven expansion in ZS cultivation necessitates sustainable development strategies as supply approaches demand. This study provides the first systematic chemical profiling of three plant parts across multiple germplasms, establishing a scientific foundation for breeding specialized cultivars. Our findings not only advance the understanding of ZS’s chemical composition but also directly support the high-value, comprehensive utilization of *Ziziphus jujuba* var. *spinosa* resources for sustainable industrial development.

## Figures and Tables

**Table 1:** Chemical constituents identified in the seeds, pulp, and leaves of ZS.

No.	t_R_ (min)	MS1 ^1^ (*m*/*z*)	Molecular Formula	Error ppm	Source			MS2 ^2^ (*m*/*z*)	Identification	Type	References
					Seed	Pulp	Leaf				
1	0.32	118.0863 [M+H]^+^	C_5_H_11_NO_2_	0.25	+	+	+	72.0443, 59.0732 [M+H−COOCH_3_]^+^, 58.0662	Valine	Amino Acids	[22]
2	0.45	117.0192 [M−H]^−^	C_4_H_6_O_4_	−1.37	+	+	+	99.9256, 73.0294 [M−H−CO_2_]^−^	Succinic acid	Organic Acids	[23]
3	0.72	179.0558 [M−H]^−^	C_6_H_12_O_6_	−1.74	+	+	+	161.0452 [M−H−H_2_O]^−^, 89.0243	*D*-Mannitol	Carbohydrate	[24]
4	0.79	138.0550 [M+H]^+^	C_7_H_7_NO_2_	0.14	+	−	−	94.0653 [M−H−C_2_H_4_O]^−^	Trigonelline	Alkaloids	[24]
5	0.80	341.1088 [M−H]^−^	C_12_H_22_O_11_	−0.38	+	−	−	179.0559, 119.0348, 89.0243	Trehalose	Carbohydrate	[24]
6	0.91	104.1071 [M+H]^+^	C_5_H_13_NO	0.86	+	+	−	60.0808 [M−H−C_2_H_4_O]^−^, 58.0661	Choline hydroxide	Alkaloids	[25]
7	1.24	118.0863 [M+H]^+^	C_5_H_11_NO_2_	0.08	+	+	+	59.0731 [M+H−COOCH_3_]^+^	Betaine	Alkaloids	[26]
8	1.28	132.1020 [M+H]^+^	C_6_H_13_NO_2_	0.38	+	+	+	86.0965 [M+H−HCOOH]^+^, 69.0610 [M+H−HCOOH-NH3]^+^	Isoleucine	Amino Acids	[27]
9	1.32	148.0605 [M+H]^+^	C_5_H_9_NO_4_	0.27	+	−	−	130.0499 [M+H−H_2_O]^+^, 102.0549, 84.0444	*L*-Glutamic acid	Amino Acids	[24]
10	1.36	279.2308 [M−H]^−^	C_18_H_32_O_2_	−7.70	+	−	−	261.3580 [M−H−H_2_O]^−^, 259.0010, 243.0622 [M−H−2H_2_O]^−^	Linoleic acid	Fatty Acids	[28]
11	1.42	243.0621 [M−H]^−^	C_9_H_12_N_2_O_6_	−0.70	+	+	+	200.0562, 110.0246, 82.0294	Uridine	Nucleotides	[24]
12	1.69	128.0352 [M−H]^−^	C_5_H_7_NO_3_	−0.70	+	−	+	82.0297	*L*-Pyroglutamic acid	-	[25]
13	1.71	191.0558 [M−H]^−^	C_7_H_12_O_6_	−1.83	+	−	+	173.0088, 85.0294	Quinic acid	Organic Acids	[27]
14	1.73	205.0353 [M−H]^−^	C_7_H_10_O_7_	−0.59	−	+	−	143.0349 [M−H−CH_3_C(OH)_2_]^−^, 87.0085 [M−H−CH_3_C(OH)_2_−C_3_H_3_O]^−^	Methylcitrate	Organic Acids	[29]
15	1.84	219.0511 [M−H]^−^	C_8_H_12_O_7_	0.23	−	+	−	111.0092 [M−H−CH_3_COOCH_2_CH_3_−H_2_O]^−^, 87.0088	Triethyl citrate	Organic Acids	[29]
16	1.90	175.1190 [M+H]^+^	C_6_H_14_N_4_O_2_	0.06	+	−	−	130.0978 [M+H−OCOH]^+^, 116.0705 [M+H−COOCH_3_]^+^, 70.0651, 60.0556	Arginine	Amino Acids	[27]
17	1.93	132.0301 [M−H]^−^	C_4_H_7_NO_4_	−1.06	+	−	−	115.0035 [M−H−OH]^−^, 71.0138	*L*-Aspartic acid	Amino Acids	[23]
18	1.93	132.1020 [M+H]^+^	C_6_H_13_NO_2_	0.38	+	+	+	86.0965, 69.0700	Leucine	Amino Acids	[23]
19	2.10	153.0192 [M−H]^−^	C_7_H_6_O_4_	−0.59	+	−	−	109.0297 [M−H−CO_2_]^−^, 108.9152, 91.5104 [M−H−CO_2_−H_2_O]^−^, 81.0345	Protocatechuic acid	Phenolic Acids	[30]
20	2.12	127.0390 [M+H]^+^	C_6_H_6_O_3_	−0.08	+	−	+	109.0284 [M+H−H_2_O]^+^, 81.0334 [M+H−H_2_O−CO]^+^, 59.6169	5-Hydroxymethylfurfural	Organic Acids	[31]
21	2.18	115.0036 [M−H]^−^	C_4_H_4_O_4_	−1.04	−	+	+	71.0137	Fumaric acid	Organic Acids	[32]
22	2.23	166.0863 [M+H]^+^	C_9_H_11_NO_2_	0.18	+	+	+	149.0597 [M+H−OH]^+^, 120.0808, 103.0544	*L*-Phenylalanine	Amino Acids	[24]
23	2.31	191.0194 [M−H]^−^	C_6_H_8_O_7_	−1.88	+	+	+	130.9985 [M−H−CH_3_C(OH)_2_]^−^, 110.6740	Citric acid	Organic Acids	[33]
24	2.31	133.0141 [M−H]^−^	C_4_H_6_O_5_	−1.20	+	+	+	115.0035 [M−H−H_2_O]^−^, 73.0001 [M−H−H_2_O−CH_2_CO]^−^, 71.0137 [M−H−H_2_O−CO_2_]^−^	Malic acid	Organic Acids	[31]
25	2.32	166.0864 [M+H]^+^	C_9_H_11_NO_2_	0.60	+	+	+	149.0595 [M+H−NH3]^+^, 120.0808 [M+H−HCOOH]^+^, 103.0538 [M+H−HCOOH−NH3]^+^	Phenylalanine	Amino Acids	[27]
26	2.32	166.1221 [M+H]^+^	C_10_H_15_NO	−3.25	+	−	−	166.0864, 121.0843 [M+H−OCOH]^+^, 103.0538	Hordenine	Alkaloids	[25]
27	2.34	136.0616 [M+H]^+^	C_5_H_5_N_5_	−1.25	+	+	+	119.9505 [M+H−OH]^+^, 109.9771, 91.9666	Adenine	Nucleotides	[24]
28	2.35	169.0140 [M−H]^−^	C_7_H_6_O_5_	−1.66	+	+	+	168.8887, 124.6000 [M−H−COOH]^−^	Gallic acid	Phenolic Acids	[34]
29	2.39	111.0088 [M−H]^−^	C_5_H_4_O_3_	0.09	+	+	+	67.0189	2-Furoic acid	Organic Acids	[25]
30	2.41	277.2172 [M−H]^−^	C_18_H_30_O_2_	−0.29	−	−	+	233.1538 [M−H−CO_2_]^−^	α-Linolenic acid	Fatty Acids	[33]
31	2.51	153.0191 [M−H]^−^	C_7_H_6_O_4_	−1.70	+	−	−	109.0291 [M−H−C_2_H_4_O]^−^	Epythricine	Phenolic Acids	[25]
32	2.54	145.0496 [M+H]^+^	C_6_H_8_O_4_	0.14	+	+	+	127.0389 [M+H−OH]^+^, 99.0440, 71.0491	5-hydroxy-4-methoxy-5,6-dihydro-2H-pyran-2-One	-	[24]
33	2.63	268.1040 [M+H]^+^	C_10_H_13_N_5_O_4_	0.00	+	−	+	136.0618	Adenosine	Nucleotides	[25]
34	2.93	116.0706 [M+H]^+^	C_5_H_9_NO_2_	0.26	+	+	+	70.0651	DL-Proline	Amino Acids	[24]
35	2.98	137.0243 [M−H]^−^	C_7_H_6_O_3_	−1.17	+	+	+	93.9087 [M−H−CO_2_]^−^, 65.0393 [M−H−CO_2_−CH_2_CH_2_]^−^	p-Hydroxybenzoic acid	Phenolic Acids	[31]
36	3.14	167.0344 [M−H]^−^	C_8_H_8_O_4_	−3.05	+	+	+	152.0120 [M−H−CH_3_]^−^	Vanillic acid	Phenolic Acids	[35]
37	3.23	341.1096 [M−H]^−^	C_12_H_22_O_11_	2.02	+	−	−	179.0563, 89.0244	Sucrose	Carbohydrate	[27]
38	3.47	368.0988 [M−H]^−^	C_16_H_19_NO_9_	0.24	+	−	−	176.0714 [M−H−Glc−CH_2_O]^−^, 158.0598 [M−H−Glc−CH_2_O−H_2_O]^−^, 144.0453 [M−H−Glc−CH_2_O−H_2_O−CH_2_]^−^	3R-*N*-glc-3-hydroxy indoleacetic acid	Amino Acids	[36]
39	3.87	188.0708 [M+H]^+^	C_11_H_9_NO_2_	1.12	+	+	+	170.0597, 146.0600, 118.0655	Trans-3-Indoleacrylic acid	-	[25]
40	3.88	153.0191 [M−H]^−^	C_7_H_6_O_4_	−1.31	+	−	−	135.9736 [M−H−H_2_O]^−^, 109.0296 [M−H−CO_2_]^−^	Protocatechuic acid	Phenolic Acids	[27]
41	4.26	370.1133 [M+H]^+^	C_16_H_19_NO_9_	−1.35	−	−	+	212.0710, 208.0606 [M+H−Glc]^+^, 190.0500 [M+H−Glc−H_2_O]^+^, 188.0708, 146.0603 [M+H−Glc−H_2_O−CO_2_]^+^	3R-1-*N*-β-*D*-glucopyranosyl-3-hydroxy-indole-3-acetic acid	Amino Acids	[37]
42	4.44	116.0706 [M+H]^+^	C_5_H_9_NO_2_	0.00	+	−	−	70.0652 [M+H−CH_2_CNH]^+^, 58.6513 [M+H−CH_2_CNH−H_2_O]^+^	Proline	Amino Acids	[32]
43	4.51	179.0350 [M−H]^−^	C_9_H_8_O_4_	0.22	+	+	+	178.9775, 134.9880 [M−H−CO_2_]^−^	Caffeic acid	Phenolic Acids	[38]
44	4.57	137.0243 [M−H]^−^	C_7_H_6_O_3_	−0.73	+	+	−	93.0345 [M−H−CO_2_]^−^	4-Hydroxybenzoic acid	Phenolic Acids	[27]
45	4.79	342.1704 [M]^+^	C_20_H_24_NO_4_	−0.35	+	+	+	297.1124 [M−OCOH]^+^, 265.0879 [M−C_6_H_6_]^+^, 58.0652	Magnoflorine	Alkaloids	[39]
46	4.98	137.0243 [M−H]^−^	C_7_H_6_O_3_	−0.78	+	+	−	108.0216 [M−H-CHO]^−^, 92.9199 [M−H−COOH]^−^	Protocatechualdehyde	Phenolic Acids	[31]
47	5.11	352.1038 [M−H]^−^	C_16_H_19_NO_8_	0.03	+	+	+	188.0714 [M−H−Glc]^−^, 146.0609 [M−H−Glc−CH_2_CNH]^−^	*N*-glc-indoleacetic acid	Amino Acids	[36]
48	5.33	181.0503 [M−H]^−^	C_9_H_10_O_4_	−1.71	+	+	+	166.0267 [M−H−CH_3_]^−^, 151.0400 [M−H−CH_2_O]^−^	Syringic acid	Phenolic Acids	[40]
49	5.53	314.1752 [M]^+^	C_19_H_24_NO_3_	−1.21	+	−	−	314.2743, 269.1175 [M−OCOH]^+^, 175.0759 [M−OCOH−C_6_H_5_O]^+^, 127.0917 [M−OCOH-C_6_H_5_O−NO−H_2_O]^+^, 107.0491	Magnocurarine	Alkaloids	[39]
50	5.77	447.1892 [M]^+^	C_23_H_29_NO_8_	−0.16	−	−	+	174.9562 [M−Glc−NO−C_6_H_7_]^+^	6-glc-Coclaurine	-	[33]
51	5.98	353.0879 [M−H]^−^	C_16_H_18_O_9_	0.20	+	+	+	352.1035, 191.0560 [M−H−CA]^−^, 173.0093 [M−H−CA−H_2_O]^−^, 161.0243 [M−H−QA]^−^, 79.0560	Chlorogenic acid	Phenolic Acids	[39]
52	6.05	289.0719 [M−H]^−^	C_15_H_14_O_6_	0.38	+	+	+	271.0603 [M−H−H_2_O]^−^, 245.0817 [M−H−CO_2_]^−^, 231.0302 [M−H−COOCH_3_]^−^, 221.0813 [M−H−CO_2_−H_2_O]^−^, 205.0504, 203.0714, 187.0400 [M−H−COOCH_3_−CO_2_]^−^, 161.0603 [M−H−H_2_O−C_6_H_6_O_2_]^−^, 125.0242, 109.0295 [M−H−H_2_O−Glc]^−^, 97.0293	(+)-Catechin/(−)-Epicatechin	Flavonoids	[41]
53	6.10	353.0874 [M−H]^−^	C_16_H_18_O_9_	−1.25	+	−	+	335.1316 [M−H−H_2_O]^−^	Cryptochlorogenic acid	Phenolic Acids	[39]
54	6.10	353.0874 [M−H]^−^	C_16_H_18_O_9_	−1.25	−	−	+	352.2861, 335.1330 [M−H−H_2_O]^−^, 135.5516 [M−H−CO_2_]^−^	Neochlorogenic acid	Phenolic Acids	[39]
55	6.15	344.1852 [M]^+^	C_20_H_26_NO_4_	−2.99	−	+	+	299.1273 [M−COOH]^+^, 175.0749, 137.0599 [M−COOH−Glc]^+^	Tembetarin	Alkaloids	[39]
56	6.45	127.0390 [M+H]^+^	C_6_H_6_O_3_	0.31	+	+	+	99.0441 [M+H−CO]^+^, 69.0334	Phloroglucinol	Phenolic Acids	[27]
57	6.49	286.1438 [M+H]^+^	C_17_H_19_NO_3_	0.00	+	+	+	269.1173 [M+H−NH_3_]^+^, 237.0910 [M+H−H_2_O]^+^, 209.0952 [M+H−H_2_O−CO]^+^, 175.0753 [M+H−NH_3_−C_6_H_6_O]^+^, 107.0491	Coclaurine	Alkaloids	[42]
58	6.82	463.0881 [M−H]^−^	C_21_H_20_O_12_	−0.26	+	+	+	301.0290 [M−H−Glc]^−^, 271.0321 [M−H−Glc−H_2_O]^−^	Quercetin-3-*O*-glucoside	Flavonoids	[38]
59	7.47	282.1128 [M + H]^+^	C_17_H_15_NO_3_	1.17	+	−	−	282.2211, 188.0713 [M+H−H_2_O−NO−OCOH]^+^	Juzirine	Alkaloids	[42]
60	7.55	220.0615 [M−H]^−^	C_11_H_11_NO_4_	−0.36	−	+	−	176.0718 [M−H−C_2_H_4_O]^−^, 69.0813	6-Hydroxy indole lactic acid	Amino Acids	[31]
61	8.25	433.1143 [M−H]^−^	C_21_H_22_O_10_	0.55	−	−	+	271.0618, 119.0349	Prunin	Flavonoids	[32]
62	8.25	193.0505 [M−H]^−^	C_10_H_10_O_4_	−0.78	−	+	+	192.9289, 133.1016 [M−H−COOCH_3_]^−^	Ferulic Acid	Phenolic Acids	[34]
63	8.45	465.1013 [M+H]^+^	C_21_H_20_O_12_	−3.2	−	+	+	303.0497 [M+H−Glc]^+^, 283.0301 [M+H−Glc−H_2_O]^+^, 85.0283	Hyperin	Flavonoids	[31]
64	8.52	449.1092 [M−H]^−^	C_21_H_22_O_11_	0.51	−	+	−	287.0562 [M−H−Glu]^−^, 269.0455 [M−H−Glu−H_2_O]^−^, 259.0614 [M−H−Glu−H_2_O−CH_2_O]^−^, 125.0247	Hovetrichoside C	Flavonoids	[37]
65	8.82	449.1083 [M+H]^+^	C_21_H_20_O_11_	1.07	−	+	+	303.0516 [M+H−Rha]^+^, 287.0549 [M+H−Glc]^+^, 85.0283, 71.0495	Quercetin-3-*O*-rjamnoside	Flavonoids	[31]
66	9.43	447.0926 [M−H]^−^	C_21_H_20_O_11_	−1.61	−	−	+	446.1139, 283.0261 [M−H−Glu]^−^, 145.0291	Astragalin	Flavonoids	[38]
67	9.56	268.1332 [M+H]^+^	C_17_H_17_NO_2_	−0.07	+	+	+	251.1066 [M+H−H_2_O]^+^, 236.0833 [M+H−CH_3_OH]^+^, 219.0804, 191.0869 [M+H−H_2_O−CH_3_COOH]^+^	Caaverine	Alkaloids	[37]
68	10.03	193.0495 [M+H]^+^	C_10_H_8_O_4_	−0.10	−	+	−	178.0264, 133.0285 [M+H−CH_3_COOH]^+^	Scopoletin	-	[31]
69	10.37	593.1508 [M−H]^−^	C_27_H_30_O_15_	−0.72	+	−	−	559.1457, 533.1267 [M−H−CH_3_COOH]^−^, 503.1179, 473.1088 [M−H−2CH_3_COOH]^−^, 457.1128, 395.0818, 383.0748, 379.0808, 353.0658, 283.0651	Vicenin II	Flavonoids	[43]
70	10.44	515.1217 [M−H]^−^	C_25_H_24_O_12_	4.27	−	−	+	515.2872, 353.0975 [M−H−Glu]^−^	Isochlorogenic acid B	-	[34]
71	10.83	137.0242 [M−H]−	C_7_H_6_O_3_	−1.31	+	+	−	109.0295, 93.0347 [M−H−CO_2_]^−^	Salicylic acid	Phenolic Acids	[40]
72	11.36	1091.5641 [M−H]^−^	C_53_H_88_O_23_	−0.25	−	−	+	1073.5580 [M−H−H_2_O]^−^, 929.5077 [M−H−Glu]^−^	3-*O*-β-*D*-glucopyranosyl-3β, 20S, 23S, 30-tetrahydroxydammar-24-en-16-on-3-*O*-β-*D*-Glucopyranosyl-(1→3)-[α-Lrhamnopyranosyl-(1→2)]-α-Larabinopyranoside	Flavonoids	[44]
73	12.45	919.2495 [M−H]^−^	C_42_H_48_O_23_	−2.01	+	−	−	757.1979 [M−H−Glc]^−^, 427.1057	6‴-(4‴-*O*-Glc)-Vanilloyspinosin	Flavonoids	[44]
74	12.52	595.1611 [M+H]^+^	C_27_H_30_O_15_	−7.85	+	−	−	433.1130 [M+H−Glu]^+^, 415.1009 [M+H−Glu−H_2_O]^+^, 367.0828, 337.0708, 313.0707, 283.0651	Isovitexin-2″-*O*-β-*D*-glucopyranoside	Flavonoids	[33]
75	12.61	595.1635 [M+H]^+^	C_27_H_30_O_15_	−3.85	+	−	−	433.1130 [M+H−Glu]^+^, 415.1022 [M+H−Glu−H_2_O]^+^, 397.0919 [M+H−Glu−2H_2_O]^+^, 379.0805 [M+H−Glu−3H_2_O]^+^, 367.0814, 337.0709, 313.0706, 283.0599	Meloside A	Flavonoids	[39]
76	12.61	595.1635 [M+H]^+^	C_27_H_30_O_15_	−3.85	+	+	+	287.0567 [M+H−Rha−Glc]^+^, 285.0402, 284.0322, 85.0286	Kaempferol-3-*O*-rutinoside	Flavonoids	[45]
77	12.73	609.1816 [M+H]^+^	C_28_H_32_O_15_	0.41	+	−	−	447.1283 [M+H−Glu]^+^, 429.1180 [M+H−Glu−H_2_O]^+^, 411.1071 [M+H−Glu−2H_2_O]^+^, 393.0971, 351.0866 [M+H−Glu−H_2_O−C_2_H_6_O_3_]^+^, 327.0863 [M+H−Glu−C_4_H_8_O_4_]^+^, 297.0757 [M+H−Glu−C_5_H_8_O_5_]^+^, 285.0748 [M+H−2−Glu]^+^, 85.0286	Isospinosin	Flavonoids	[39]
78	12.77	433.1147 [M+H]^+^	C_21_H_20_O_10_	4.16	+	−	−	415.1037 [M+H−H_2_O]^+^, 397.0919 [M+H−2H_2_O]^+^, 379.0822 [M+H−3H_2_O]^+^, 367.0814, 337.0712 [M+H−2H_2_O−CH_3_COOH]^+^, 313.0708 [M+H−2CH_3_COOH]^+^, 283.0613	Vitexin	Flavonoids	[37]
79	12.82	725.1926 [M−H]^−^	C_32_H_38_O_19_	−1.13	+	+	+	593.1478 [M−H−Glu]^−^, 575.1392 [M-H−Glu−H_2_O]^−^, 285.0404, 284.0328	Camellianin B	Flavonoids	[37]
80	12.83	282.1489 [M+H]^+^	C_18_H_19_NO_2_	0.04	+	+	−	282.1489, 266.1258, 265.1223 [M+H−H_2_O]^+^, 250.0991 [M+H−CH_3_OH]^+^, 234.1039, 191.1497	*N*-nornuciferine	Alkaloids	[37]
81	12.83	609.1820 [M+H]^+^	C_28_H_32_O_15_	0.92	+	−	−	489.1374, 447.1283 [M+H−Glu]^+^, 429.1184 [M+H−Glu−H_2_O]^+^, 411.1076 [M+H−Glu−2H_2_O]^+^, 393.0975, 381.0971, 351.0868 [M+H−Glu−H_2_O−C_2_H_6_O_3_]^+^, 327.0866 [M+H−Glu−C_4_H_8_O_4_]^+^, 297.0766 [M+H−Glu−C_5_H_8_O_5_]^+^, 285.0757 [M+H−2−Glu]^+^	Spinosin	Flavonoids	[37]
82	12.87	433.1137 [M+H]^+^	C_21_H_20_O_10_	1.89	+	−	−	415.1037 [M+H−H_2_O]^+^, 397.0919 [M+H−2H_2_O]^+^, 379.0839 [M+H−3H_2_O]^+^, 367.0814, 337.0705 [M+H−2H_2_O−CH_3_COOH]^+^, 313.0714 [M+H−2CH_3_COOH]^+^, 283.0610	Isovitexin	Flavonoids	[37]
83	12.89	447.1284 [M+H]^+^	C_22_H_22_O_10_	−0.40	+	+	−	429.1183 [M+H−H_2_O]^+^, 411.1060 [M+H−2H_2_O]^+^, 393.0972 [M+H−3H_2_O]^+^, 351.0856, 327.0875 [M+H−2CH_3_COOH]^+^, 297.0762 [M+H−2CH_3_COOH−CO]^+^, 281.0668 [M+H−2CH_3_COOH−CO−CH_3_]^+^	Swertisin	Flavonoids	[37]
84	12.90	607.1665 [M−H]^−^	C_28_H_32_O_15_	−0.56	+	−	−	299.0244 [M−H−Rha−Glc]^−^	Diosmin	Flavonoids	[27]
85	13.03	609.1821 [M+H]^+^	C_28_H_32_O_15_	1.12	+	−	−	489.1410 [M+H−2CH_3_COOH]^+^, 447.1286 [M+H−Glu]^+^, 429.1185 [M+H−Glu−H_2_O]^+^, 327.0861	Genistein-7-*O*-glucuronide	Flavonoids	[33]
86	13.09	609.1468 [M−H]^−^	C_27_H_30_O_16_	1.13	+	+	+	301.0349 [M−H−Glc−Rha]^−^, 178.9975	Rutin	Flavonoids	[45]
87	13.11	485.3113 [M+H]^+^	C_27_H_40_N_4_O_4_	−2.02	+	−	+	115.1312, 114.1278	Mucronine J	Alkaloids	[42]
88	13.23	795.4533 [M−H]^−^	C_42_H_68_O_14_	−0.39	−	+	+	749.4515 [M−H−COOH]^−^	Jujubasaponin VI	Terpenoids	[44]
89	13.24	759.2126 [M+H]^+^	C_36_H_38_O_18_	−0.66	+	+	+	639.1703 [M+H−2CH_3_COOH]^+^, 429.1180, 381.0974,	6‴-vanilloylspinosin	Flavonoids	[33]
90	13.26	771.2130 [M+H]^+^	C_37_H_38_O_18_	−0.10	+	−	+	651.1766 [M+H−2CH_3_COOH]^+^, 609.1599 [M+H−Glu]^+^, 337.0719, 145.0282	Isovitexin-2″-*O*-(6-feruloyl)-glucopyranoside	Flavonoids	[33]
91	13.28	729.2051 [M+H]^+^	C_35_H_36_O_17_	3.51	+	−	−	609.1555 [M+H−2CH_3_COOH]^+^, 447.1273, 429.1181	6‴-*O*-p-Hydroxybenzoylspinosin	Flavonoids	[33]
92	13.28	944.2814 [M+H]+	C_44_H_49_NO_22_	−0.49	+	−	−	824.2395 [M+H−CH_3_COOH]^+^, 327.0864, 297.0758	6‴-*O*-(3-Glc-indole-acetyl)spinosin	Alkaloids	[39]
93	13.28	945.2865 [M+H]^+^	C_44_H_50_NO_22_	−3.41	+	−	−	824.2395 [M+H−Glu]^+^, 429.1182, 285.0759	6‴-*O*-(3-Indoleglucoside-Acetyl)spinosin	Alkaloids	[33]
94	13.30	741.1977 [M+H]^+^	C_36_H_36_O_17_	−6.57	+	−	−	433.11353 [M+H−Glu]^+^, 337.07095, 313.07071, 147.04408	Isovitexin-2′’-*O*-(6-p-Coumaroyl)Glucopyranoside	Flavonoids	[42]
95	13.36	815.2410 [M+H]^+^	C_39_H_42_O_19_	2.05	+	−	−	695.2031, 609.1816 [M+H−sinapoyl]^+^, 447.1293, 429.1179 [M+H−sinapoyl−Glc−H_2_O]^+^, 411.1063 [M+H−siapoyl−Glc−2H_2_O]^+^, 393.0981 [M+H−sinapoyl−Glc−3H_2_O]^+^, 351.0850, 327.0864, 297.0758, 207.0652	6‴-Sinapoylspinosin	Flavonoids	[39]
96	13.42	755.2183 [M+H]^+^	C_37_H_38_O_17_	0.20	+	−	−	635.1754 [M+H−2CH_3_COOH]^+^, 447.1288, 327.0865	6‴-p-Coumaroylspinosin	Flavonoids	[44]
97	13.42	785.2286 [M+H]^+^	C_38_H_40_O_18_	−0.13	+	−	−	665.1857 [M+H−2CH_3_COOH]^+^, 605.1653, 489.1408	6‴-Feruloylspinosin	Flavonoids	[36]
98	13.44	873.3129 [M+H]^+^	C_43_H_52_O_19_	−5.29	+	−	−	855.3099 [M+H−H_2_O]^+^, 735.2687 [M+H−H_2_O−2CH_3_COOH]^+^, 447.1285	6‴-Dihydrophaseoylspinosin	Flavonoids	[36]
99	13.51	785.2272 [M+H]^+^	C_38_H_40_O_18_	−2.00	+	−	−	665.1873 [M+H−2CH_3_COOH]^+^, 447.1285, 177.0548	6‴-Feruloylisospinosin	Flavonoids	[36]
100	13.78	869.2864 [M−H]^−^	C_43_H_50_O_19_	−1.05	+	−	−	839.2766 [M−H−CH_2_O]^−^, 607.1705, 427.1036	6‴-(-)-Phaseoylspinosin	Flavonoids	[33]
101	13.95	961.2761 [M−H]^−^	C_48_H_50_O_21_	−1.10	+	−	−	943.2663 [M−H−H_2_O]^−^, 931.2672 [M−H−2CH_3_]^−^	6″-p-coumaroyl-6‴-sinapoylspinosin	Flavonoids	[33]
102	14.00	535.3284 [M+H]^+^	C_31_H_42_N_4_O_4_	1.03	+	−	−	149.1153, 148.1122	Sanjoinine A	Alkaloids	[42]
103	14.00	1121.3337 [M+H]^+^	C_54_H_58_NO_25_	−2.96	+	+	+	1102.3140 [M+H−H_2_O]^+^, 940.2723 [M+H−Glc]^+^, 327.0863	6″-*O*-(3-glc-indole-acetyl)-6‴-feruloylspinosin	Alkaloids	[46]
104	14.10	632.3815 [M+H]^+^	C_36_H_49_N_5_O_5_	1.41	+	−	+	344.1968, 289.1912, 148.1121	Amphibin D	Alkaloids	[37]
105	14.54	301.0354 [M−H]^−^	C_15_H_10_O_7_	−0.03	+	+	+	301.2016 [M−H]^−^, 300.0259, 273.0399 [M−H−CO]^−^, 257.1294 [M−H−CO−H_2_O]^−^, 178.0036	Quercetin	Flavonoids	[38]
106	14.43	1205.5936 [M−H]^−^	C_58_H_94_O_26_	−0.57	+	+	+	1055.3833 [M−H−Rha−H_2_O]^−^, 911.3578 [M−H−Rha−Glc]^−^, 749.2642 [M−H−Rha−2Glc]^−^, 603.2457 [M−H−Rha−2Glc−Xyl]^−^, 455.2535	Jujuboside A	Terpenoids	[33]
107	14.56	933.2449 [M−H]^−^	C_46_H_46_O_21_	−1.02	+	−	−	783.2073, 663.1733, 577.1345, 235.0812	6″-feruloyl-6‴-vanillylspinosin	Flavonoids	[44]
108	14.80	484.3199 [M−H]^−^	C_30_H_45_O_5_	0.97	−	+	+	423.3266 [M−H−CH_3_C(OH)_2_]^−^	Epiceanothic acid	Terpenoids	[47]
109	14.98	285.0406 [M−H]^−^	C_15_H_10_O_6_	0.46	−	+	+	284.1950, 151.0036 [M−H−C_4_H_4_O_4_−H_2_O]^−^	Kaempferol	Flavonoids	[34]
110	15.20	455.3519 [M+H]^+^	C_30_H_46_O_3_	−0.15	+	+	+	409.3472, 189.1637	Betulonic acid	Terpenoids	[48]
111	15.24	391.2837 [M+H]^+^	C_24_H_38_O_4_	−0.46	+	−	−	167.1049, 149.1322	Bis(2-ethylhexyl) phthalate	-	[24]
112	15.27	959.2600 [M−H]^−^	C_48_H_48_O_21_	−1.58	+	−	−	783.2116 [M−H−Glu]^−^	6″,6‴-diferuloylspinosin	Flavonoids	[44]
113	15.49	385.1633 [M+H]^+^	C_22_H_24_O_6_	−3.27	−	+	−	285.0781	Schisandrin C	-	[33]
114	15.53	329.2331 [M−H]^−^	C_18_H_34_O_5_	−0.70	+	+	+	229.1440, 211.1340	9,12,13-Trihydroxy-11-octadecenoic acid	Fatty Acids	[49]
115	15.54	473.3625 [M+H]^+^	C_30_H_48_O_4_	−0.11	+	+	+	455.3545 [M+H−H_2_0]^+^, 437.3425 [M+H−2H_2_0]^+^, 409.3479, 391.2832	Corosolic acid	Terpenoids	[50]
116	15.84	1043.5425 [M−H]^−^	C_52_H_84_O_21_	−0.72	+	+	+	911.5001 [M−H−Xyl]^−^, 893.4887, 749.4475 [M−H−Xyl−Glu]^−^, 603.3878 [M−H−Xyl−Glu−Xyl]^−^	Jujuboside B	Terpenoids	[51]
117	16.18	254.2246 [M−H]^−^	C_16_H_31_O_2_	−1.97	−	+	−	255.0879	Hexadecanoic acid	Fatty Acids	[47]
118	16.20	1085.5529 [M−H]^−^	C_54_H_86_O_22_	−0.86	+	−	−	1043.5425 [M−H−CH_2_CO]^−^, 1025.5322 [M−H−CH_3_COOH]^−^, 765.4409, 749.4477	Acetyl-jujuboside B	Terpenoids	[33]
119	16.72	474.3709 [M+H]^+^	C_30_H_49_O_4_	1.22	−	+	+	473.3645, 409.3476 [M+H−C_5_H_5_]^+^	2α-hydroxyursolic acid	Terpenoids	[47]
120	16.94	501.3225 [M−H]^−^	C_30_H_46_O_6_	0.62	−	+	+	501.3222 [M−H]^−^	27-Hydroxyceanothic acid	Terpenoids	[47]
121	17.37	501.3218 [M−H]^−^	C_30_H_46_O_6_	−0.76	+	+	+	471.3110 [M−H−CH_2_O]^−^, 439.3212 [M−H−CH_3_C(OH)_2_]^−^	24-Hydroxyceanothic acid	Terpenoids	[47]
122	17.48	485.3272 [M−H]^−^	C_30_H_46_O_5_	−0.12	−	−	+	423.3313	Ceanothic acid	Terpenoids	[36]
123	17.81	313.2382 [M−H]^−^	C_18_H_34_O_4_	−0.86	+	+	−	183.1384, 129.0920	(±)9(10)-Dihome	Organic Acids	[24]
124	17.93	313.2384 [M−H]^−^	C_18_H_34_O_4_	−0.19	+	+	−	201.1133	(±)12(14)-Dihome	Organic Acids	[24]
125	18.05	320.2590 [M+H]^+^	C_20_H_33_NO_2_	1.94	+	−	−	302.2493 [M+H−H_2_O]^+^	Mestanolone	Alkaloids	[25]
126	18.30	457.3682 [M+H]^+^	C_30_H_48_O_3_	1.18	−	−	+	439.3756 [M+H−H_2_O]^+^, 179.1444, 135.1173	Ursolic acid	Terpenoids	[31]
127	18.42	485.3272 [M−H]^−^	C_30_H_46_O_5_	0.00	+	+	+	467.3186 [M−H−H_2_O]^−^, 423.3261, 60.9930	Emmolic Acid	Terpenoids	[36]
128	18.96	295.2277 [M−H]^−^	C_18_H_32_O_3_	−0.71	+	+	+	277.2171 [M−H−H_2_O]^−^, 171.1023	10E,12Z-octadecadienoic acid	Fatty Acids	[24]
129	19.01	281.2482 [M−H]^−^	C_18_H_34_O_2_	−1.42	+	+	+	281.0876 [M−H]^−^	Oleic acid	Fatty Acids	[47]
130	19.07	235.1694 [M+H]^+^	C_15_H_22_O_2_	0.60	+	+	+	179.1071	3,5-Di-tert-butyl-4-hydroxybenzaldehyde	-	[25]
131	19.61	469.3321 [M−H]^−^	C_30_H_46_O_4_	−0.45	+	+	+	451.3235 [M−H−H_2_O]^−^, 423.8594 [M−H−CO−H_2_O]^−^	Zizyberanalic acid	Terpenoids	[44]
132	20.12	255.2330 [M−H]^−^	C_16_H_32_O_2_	0.31	+	−	+	219.8454 [M−H−2H_2_O]^−^	Palmitic acid	Fatty Acids	[52]
133	21.00	471.3479 [M−H]^−^	C_30_H_48_O_4_	−0.23	−	−	+	471.3479 [M−H]^−^	Alphitolic acid	Terpenoids	[39]
134	21.27	282.2548 [M−H]^−^	C_18_H_35_O_2_	−5.77	−	−	+	283.2642	Octadecanoic acid	Fatty Acids	[47]
135	21.63	338.3418 [M+H]^+^	C_22_H_43_NO	0.09	+	+	+	233.2274, 163.1481, 97.1012	Erucamide	Fatty Acids	[24]
136	21.85	256.2635 [M+H]^+^	C_16_H_33_NO	0.00	+	+	+	88.0755	Hexadecanamide	Fatty Acids	[24]
137	21.90	455.3531 [M−H]^−^	C_30_H_48_O_3_	0.00	+	+	+	455.0169, 409.3474 [M−H−COOH]^−^, 407.5913, 217.1960, 191.1793, 121.1010	Betulinic acid	Terpenoids	[39]
138	22.20	282.2792 [M+H]^+^	C_18_H_35_NO	0.35	+	+	−	265.2527 [M+H−OH]^+^, 114.0914, 69.0698, 57.0698	Oleamide	Fatty Acids	[24]
139	22.40	453.3371 [M−H]^−^	C_30_H_46_O_3_	−0.62	+	+	+	451.3217, 255.2330, 180.9730	Betulonic acid	Terpenoids	[36]
140	22.96	599.3210 [M−H]^−^	C_27_H_53_O_12_P	1.32	−	+	+	315.0497, 283.2645, 241.0124, 152.9961	1-octadecanoyl-sn-glycero-3-phospho-(1′-myo-inositol)	Fatty Acids	[39]
141	23.15	452.3257 [M−H]^−^	C_30_H_45_O_3_	−8.71	+	−	+	409.3156 [M−H−CH_3_CO]^−^	Oleanolic acid	Terpenoids	[47]
142	23.69	265.1478 [M−H]^−^	C_12_H_26_O_4_S	−0.38	+	+	−	96.9599	(dodecyloxy)Sulfonic acid	-	[25]
143	24.86	595.2882 [M−H]^−^	C_27_H_49_O_12_P	−1.23	+	+	+	595.0358, 279.2332, 241.0115	1-(9Z,12Z-octadecadienoyl)-glycero-3-phospho-(1′-myo-inositol)	Fatty Acids	[39]

^1^ MS1: Mass Spectrometry; ^2^ MS2: Tandem Mass Spectrometry.

## Data Availability

The original contributions presented in this study are included in the article/Supplementary Materials. Further inquiries can be directed to the corresponding author.

## Supplementary Materials

The following supporting information can be downloaded at: https://www.mdpi.com/article/10.3390/molecules30224470/s1.

## Appendix A

**Table A1:** Results of methodological validation for the quantitative analysis of major chemical composition in the seeds, pulp, and leaves of ZS.

Part	NO.	Compounds	Regression Equation	R^2^	Linear Range (mg/mL)	Precision (RSD%; *n* = 6)	Repeatability (RSD%; *n* = 6)	Stability (RSD%; *n* = 6)	Recovery	
									Mean	RSD%
Seed	1	Cryptochlorogenic acid	y = 181.7x − 8.2576	0.999	4.80 × 10^−5^–9.60 × 10^−4^	0.63	0.29	2.93	98.48	0.63
	2	Caffeic acid	y = 331.74x + 16.201	0.999	1.20 × 10^−3^–2.40 × 10^−2^	0.57	0.52	2.82	98.01	0.01
	3	Spinosin	y = 169.99x + 9.557	1.000	9.90 × 10^−4^–1.98 × 10^−2^	0.22	0.07	0.97	99.28	1.08
	4	6‴-Feruloylspinosin	y = 142.31x + 7.0431	0.999	1.09 × 10^−3^–2.18 × 10^−2^	0.31	0.03	0.98	99.14	0.85
	5	Jujuboside A	y = 20.72x − 44.533	0.999	9.60 × 10^−4^–1.92 × 10^−2^	0.37	0.21	2.99	98.51	0.32
	6	Jujuboside B	y = 16.945x − 37.072	0.999	9.50 × 10^−4^–1.90 × 10^−2^	0.44	0.32	2.64	99.20	1.05
Pulp	7	Citric acid	y = 259.6x + 95.312	0.999	1.48 × 10^−3^–4.44 × 10^−2^	1.82	2.91	1.45	99.55	2.05
	8	Gallic acid	y = 194.22x − 4.8952	0.999	1.41 × 10^−5^–1.41 × 10^−3^	0.35	2.64	2.08	100.79	0.93
	9	Catechin	y = 85.116x + 10.774	0.999	1.34 × 10^−5^–1.34 × 10^−3^	0.43	2.34	2.03	100.11	0.50
	2	Caffeic acid	y = 484.79x + 11.078	0.999	1.49 × 10^−6^–2.97 × 10^−4^	0.38	2.78	2.36	102.12	0.38
	10	Ferulic Acid	y = 439.01x + 12.786	0.999	1.54 × 10^−6^–3.07 × 10^−4^	0.57	1.42	1.44	100.75	0.97
	11	Rutin	y = 91.964x + 13.828	0.999	1.34 × 10^−5^–1.34 × 10^−3^	0.47	1.96	2.44	99.76	1.35
	12	Quercetin-3-*O*-glucoside	y = 129x + 6.4427	0.999	7.29 × 10^−6^–7.29 × 10^−4^	0.42	2.95	2.78	102.32	2.06
Leaf	7	Citric acid	y = 22.597x − 28.666	0.999	2.59 × 10^−4^–2.59 × 10^−3^	1.98	2.98	2.92	101.40	2.97
	13	Neochlorogenic acid	y = 16.42x + 3.6476	0.999	5.58 × 10^−6^–1.12 × 10^−4^	0.89	2.97	2.03	101.10	2.55
	9	Catechin	y = 68.325x + 2.3997	0.999	1.97 × 10^−5^–7.88 × 10^−4^	0.34	2.89	2.90	102.12	0.66
	2	Caffeic acid	y = 62.608x + 8.4197	0.999	9.85 × 10^−6^–3.94 × 10^−4^	0.54	2.99	2.59	100.50	2.85
	11	Rutin	y = 1371.3x + 1479	0.999	5.50 × 10^−3^–5.50 × 10^−2^	0.43	2.92	2.73	99.86	2.64
	12	Quercetin-3-*O*-glucoside	y = 92.852x + 9.7727	0.999	2.04 × 10^−4^–2.04 × 10^−3^	0.46	2.34	2.31	100.14	2.71
	14	Kaempferol-3-*O*-rutoside	y = 108.22x + 13.042	0.999	5.65 × 10^−5^–2.26 × 10^−3^	0.15	2.99	2.74	102.98	0.80
	15	Isochlorogenic acid B	y = 1898.4x + 29.204	1.000	1.08 × 10^−6^–1.08 × 10^−2^	2.95	2.32	2.72	97.50	1.02
	16	Astragalin	y = 35.406x + 2.4954	0.999	1.09 × 10^−5^–4.36 × 10^−4^	2.94	2.82	2.86	101.46	2.66
	17	Quercetin	y = 100.09x − 122.13	0.999	1.12 × 10^−4^–1.68 × 10^−3^	2.96	2.71	2.72	101.74	0.47
	5	Jujuboside A	y = 46.259x − 55.494	0.999	1.18 × 10^−4^–2.35 × 10^−3^	2.21	2.94	2.09	100.73	1.90
	6	Jujuboside B	y = 29.055x − 28.964	0.999	1.07 × 10^−4^–2.13 × 10^−3^	1.41	2.74	2.51	100.76	2.01
	18	Kaempferol	y = 102.2x − 81.167	0.999	1.03 × 10^−4^–4.12 × 10^−3^	2.21	2.69	2.45	102.65	0.83

**Table A2:** Contents of major chemical composition in seeds of different ZS germplasms (*n* = 3; mg/g).

No.	Cryptochlorogenic Acid	Caffeic Acid	Spinosin	6‴-Feruloyl-Spinosyn	Jujuboside A	Jujuboside B	Total Content
ZS1	0.1062	0.7112	1.2886	2.3730	0.3141	0.1938	4.9869
ZS2	0.0727	0.6286	0.7910	2.2690	0.4826	0.1521	4.3960
ZS3	0.1646	0.9589	1.4800	5.0200	0.6344	0.1938	8.4517
ZS4	0.0725	1.0770	1.8418	3.9023	0.6084	0.1942	7.6962
ZS5	0.0587	0.5209	0.8595	1.8880	0.3145	0.1618	3.8034
ZS6	0.1395	0.6972	1.3440	3.6170	0.7288	0.1564	6.6829
ZS7	0.0565	0.6188	0.8110	1.6747	0.2615	0.1494	3.5719
ZS8	0.0623	0.6414	1.0281	2.5806	0.2980	0.1250	4.7354
ZS9	0.1281	0.5376	0.8448	2.8698	0.4869	0.1448	5.0120
ZS10	0.1466	0.5632	1.1062	2.8243	0.6565	0.1462	5.4430
ZS11	0.0741	0.8066	0.7994	2.9534	0.3466	0.1328	5.1129
ZS12	0.0537	0.6482	0.9176	3.7911	0.4211	0.1499	5.9816
ZS13	0.0602	0.7359	1.3451	4.6438	0.5297	0.1584	7.4731
ZS14	0.0909	0.6125	1.4105	2.7143	0.5073	0.1621	5.4976
ZS15	0.1818	0.9821	1.4245	3.8416	0.6744	0.1721	7.2765
ZS16	0.1577	0.7805	1.1325	4.0440	0.5537	0.1585	6.8269
ZS17	0.1129	1.0943	0.9379	3.2048	0.4883	0.1541	5.9923
ZS18	0.1069	0.8839	0.7133	3.7788	0.4744	0.1578	6.1151
ZS19	0.1139	0.9753	0.7203	3.9951	0.4863	0.1590	6.4499
ZS20	0.0394	0.5856	0.7227	1.4580	0.2964	0.1523	3.2544
ZS21	0.0639	0.5653	0.3413	1.5143	0.2396	0.1308	2.8552
ZS22	0.1498	1.4030	0.9837	5.5312	0.7107	0.1792	8.9576
ZS23	0.0592	0.6142	0.9316	2.6552	0.4238	0.1551	4.8391
ZS24	0.1591	2.1300	1.6016	5.1558	0.9615	0.1695	10.1775
ZS25	0.0801	0.4998	0.6658	2.0807	0.2968	0.1283	3.7515
ZS26	0.0497	0.1842	0.4621	2.2039	0.3340	0.1410	3.3749
CV(%)	43.38	46.62	35.32	36.00	36.45	11.98	

**Table A3:** Contents of major chemical composition in pulp of different ZS germplasms (*n* = 3; mg/g).

No.	Citric Acid	Gallic Acid	Catechin	Caffeic Acid	Ferulic Acid	Rutin	Quercetin-3-*O*-Glucoside	Total Content
ZS1	20.4395	0.1283	0.0878	0.0069	0.0042	0.1586	0.0125	20.8378
ZS2	12.6134	0.0907	0.1239	0.0146	0.0023	0.1455	0.0124	13.0028
ZS3	18.0934	0.1619	0.1485	0.0181	0.0006	0.3071	0.0148	18.7444
ZS4	7.2853	0.0814	0.0738	0.0018	0.0025	0.0533	0.0091	7.5072
ZS5	10.0898	0.2129	0.1340	0.0030	0.0032	0.1538	0.0093	10.606
ZS6	14.6059	0.1527	0.1240	0.0136	0.0011	0.3270	0.0137	15.238
ZS7	5.2575	0.0778	0.0406	0.0089	0.0007	0.0855	0.0063	5.4773
ZS8	13.9532	0.1101	0.1133	0.0150	0.0009	0.1079	0.0090	14.3094
ZS9	16.7025	0.1355	0.1187	0.0111	0.0014	0.2884	0.0101	17.2677
ZS10	13.7535	0.0947	0.1371	0.0131	0.0007	0.3081	0.0114	14.3186
ZS11	18.1177	0.1323	0.0413	0.0175	0.0081	0.1208	0.0062	18.4439
ZS12	17.0808	0.0799	0.0936	0.0078	0.0076	0.2349	0.0025	17.5071
ZS13	14.4683	0.0714	0.0739	0.0017	0.0075	0.2126	0.0091	14.8445
ZS14	17.0504	0.1662	0.1129	0.0148	0.0028	0.1450	0.0063	17.4984
ZS15	13.0702	0.0674	0.1149	0.0095	0.0018	0.2273	0.0059	13.497
ZS16	8.8191	0.0326	0.1123	0.0017	0.0024	0.1333	0.0114	9.1128
ZS17	3.4939	0.0597	0.1281	0.0109	0.0027	0.0834	0.0107	3.7894
ZS18	2.4508	0.0464	0.0983	0.0056	0.0011	0.0428	0.0103	2.6553
ZS19	4.1632	0.1063	0.0947	0.0106	0.0021	0.0596	0.0120	4.4485
ZS20	4.9013	0.1578	0.0803	0.0163	0.0022	0.2230	0.0112	5.3921
ZS21	6.9057	0.1775	0.0510	0.0077	0.0070	0.0678	0.0103	7.227
ZS22	12.9828	0.1075	0.0840	0.0112	0.0036	0.1039	0.0122	13.3052
ZS23	1.4692	0.1441	0.0815	0.0041	0.0028	0.0831	0.0112	1.796
ZS24	10.1719	0.1142	0.1165	0.0087	0.0023	0.2351	0.0131	10.6618
ZS25	6.5215	0.1217	0.0835	0.0038	0.0023	0.0653	0.0108	6.8089
ZS26	2.2088	0.2219	0.0586	0.0090	0.0025	0.0280	0.0081	2.5369
CV(%)	53.70	41.11	30.44	52.41	74.87	58.92	28.00	

**Table A4:** Contents of major chemical composition in leaves of different ZS germplasms (*n* = 3; mg/g).

No.	Citric Acid	Neochlorogenic Acid	Catechin	Caffeic Acid	Rutin	Quercetin-3-*O*-Glucoside	Kaempfer-ol-3-*O*-Rutoside	Isochlorogenic Acid B	Astragalin	Querce-Tin	Jujuboside A	**Jujuboside B**	**Kaempferol**	**Total** **Content**
ZS1	0.5709	0.0275	0.0897	0.0308	11.1865	0.3571	0.0921	0.0261	0.0693	0.2753	0.6068	0.3064	0.3229	13.9614
ZS2	0.5089	0.0157	0.0424	0.0457	7.5060	0.6052	0.3063	0.0386	0.0610	0.1344	0.5541	0.2446	0.3106	10.3735
ZS3	0.9195	0.0046	0.1344	0.0489	5.7306	0.5200	0.2046	2.2140	0.0849	0.1150	0.4081	0.2854	0.4080	11.078
ZS4	0.7810	0.0478	0.0300	0.0709	10.0390	0.5569	0.2977	1.7245	0.0544	0.1227	0.3168	0.2025	0.2656	14.5098
ZS5	0.5405	0.0155	0.1039	0.0157	3.0968	0.6526	0.3868	1.5529	0.1065	0.0967	0.2268	0.1099	0.4777	7.3823
ZS6	1.0364	0.0133	0.0379	0.1302	18.8823	0.6733	0.7043	0.0416	0.1379	0.1540	0.4177	0.4441	0.4645	23.1375
ZS7	0.4347	0.0107	0.0150	0.0429	2.8404	0.3405	0.0598	1.2509	0.0439	0.0910	0.5921	0.1577	0.2105	6.0901
ZS8	0.5221	0.0106	0.0248	0.0526	5.4711	0.2564	0.1500	0.7973	0.0143	0.1135	0.1440	0.1711	0.3087	8.0365
ZS9	0.8177	0.0415	0.0281	0.0620	13.3891	0.5901	0.3803	0.1045	0.0529	0.1316	0.1063	0.3690	0.9751	17.0482
ZS10	0.5645	0.0099	0.0659	0.0830	9.6662	0.4719	0.3710	0.0332	0.0844	0.1036	0.2724	0.2821	0.3268	12.3349
ZS11	0.8775	0.0410	0.0605	0.0126	17.7091	0.3422	0.1191	0.0306	0.0461	0.2499	0.8472	0.1333	0.4671	20.9362
ZS12	0.4080	0.0106	0.0088	0.0391	11.0067	0.3394	0.2339	0.0024	0.0312	0.1687	0.4293	0.3416	0.4284	13.4481
ZS13	0.5045	0.0136	0.0176	0.0520	12.2706	0.3904	0.2340	0.0119	0.0373	0.2217	0.5657	0.4504	0.5333	15.303
ZS14	0.5120	0.0122	0.0102	0.0955	5.3304	0.4209	0.1608	2.0755	0.0403	0.1677	0.2860	0.2943	0.3945	9.8003
ZS15	0.7865	0.0354	0.0129	0.0732	12.2471	0.5399	0.3339	0.0844	0.0909	0.1405	0.1028	0.2935	0.9070	15.648
ZS16	0.5200	0.0141	0.0252	0.0266	11.1758	0.3514	0.1402	0.0164	0.0214	0.1681	0.4261	0.2741	0.3892	13.5486
ZS17	0.9237	0.0296	0.0089	0.0769	10.3866	0.6467	0.2408	2.2678	0.0505	0.2046	0.4654	0.4048	0.4513	16.1576
ZS18	0.9343	0.0261	0.0382	0.0915	8.7731	0.7476	0.2424	3.1403	0.0495	0.2369	0.3470	0.3817	0.5601	15.5687
ZS19	0.5011	0.0211	0.0270	0.0514	5.8081	0.5051	0.1189	2.1670	0.0380	0.1515	0.5541	0.2828	0.3797	10.6058
ZS20	1.0655	0.0296	0.0109	0.1216	14.7244	0.8187	0.4353	2.1744	0.1644	0.3637	0.1360	0.4444	1.5576	22.0465
ZS21	0.6110	0.0220	0.0176	0.0498	5.3191	0.3270	0.1322	2.1690	0.0293	0.1277	0.2187	0.3844	0.6718	10.0796
ZS22	0.3820	0.0133	0.0187	0.0363	3.1398	0.3311	0.0652	1.3516	0.0104	0.1034	0.3316	0.1694	0.2585	6.2113
ZS23	0.6581	0.0120	0.0358	0.0180	7.8348	0.4324	0.3036	1.4796	0.0425	0.2302	0.6041	0.2018	0.2860	12.1389
ZS24	0.9091	0.0458	0.0094	0.0655	16.1573	0.5281	0.3047	0.0699	0.0793	0.1554	0.1221	0.3515	1.0696	19.8677
ZS25	0.4940	0.0239	0.0107	0.0611	6.1704	0.3545	0.2018	1.9754	0.0509	0.1355	0.3014	0.1707	0.2315	10.1818
ZS26	0.8259	0.0216	0.0088	0.0500	9.0060	0.3979	0.1736	1.6415	0.0248	0.1255	0.1866	0.2935	0.4068	13.1625
CV(%)	30.81	55.73	93.65	51.23	46.99	30.71	57.13	92.25	62.53	39.21	52.26	35.05	61.45	13.9614
